# Synergistic Binary Fe–Co Nanocluster Supported on Defective Tungsten Oxide as Efficient Oxygen Reduction Electrocatalyst in Zinc‐Air Battery

**DOI:** 10.1002/advs.202104237

**Published:** 2021-12-01

**Authors:** Qinglin Han, Ximeng Zhao, Yuhong Luo, Lanlan Wu, Shujuan Sun, Jingde Li, Yanji Wang, Guihua Liu, Zhongwei Chen

**Affiliations:** ^1^ Hebei Provincial Key Laboratory of Green Chemical Technology and Highly Efficient Energy Saving Tianjin Key Laboratory of Chemical Process Safety National‐Local Joint Engineering Laboratory for Energy Conservation of Chemical Process Integration and Resources Utilization School of Chemical Engineering and Technology Hebei University of Technology Tianjin 300130 P. R. China; ^2^ Department of Chemical Engineering University of Waterloo Waterloo ON N2L 3G1 Canada

**Keywords:** oxygen reduction reaction, oxygen‐deficient support, synergistic effect, zinc–air batteries

## Abstract

Rational design of metal oxide supported non‐precious metals is essential for the development of stable and high‐efficiency oxygen reduction reaction (ORR) electrocatalysts. Here, an efficient ORR catalyst consisting of binary Fe/Co nanoclusters supported by defective tungsten oxide and embedded N‐doped carbon layer (NC) with a 3D ordered macroporous architecture (3DOM Fe/Co@NC‐WO_2−_ *_x_*) is developed. The oxygen deficient 3DOM WO_2−_ *_x_* not only serves as a porous and stable support, but also enhances the conductivity and ensures good dispersion of the binary Fe/Co nanocluster, benefiting its ORR catalytic activity. Theoretical calculation shows that there exists a synergistic effect of electron transfer from Fe to Co in the supported binary Fe/Co cluster, promoting the ORR reaction energetics. Accordingly, the 3DOM Fe/Co@NC‐WO_2−_ *_x_* catalyst exhibits excellent ORR activity in alkaline medium with a half wave potential (*E*_1/2_) of 0.87 V higher than that of Pt/C (0.85 V). The zinc–air batteries assembled by 3DOM Fe/Co@NC‐WO_2−_ *_x_* cathode deliver a higher power density and specific capacity than that of Pt/C. A new strategy of combining synergistic binary‐metal nanoclusters and conductive metal oxide support design is provided here to develop efficient and durable ORR electrocatalyst.

## Graphical abstract

Efficient oxygen reduction reaction (ORR) catalyst consisting of binary Fe/Co nanoclusters supported by defective tungsten oxide and embedded N‐doped carbon layer with a 3D ordered macroporous architecture is developed. The oxygen deficient 3DOM WO_2−_ *_x_* serves as a porous and stable support, enhances the conductivity, and ensures good dispersion of the binary Fe/Co nanocluster, benefiting its ORR catalytic activity.

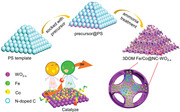

## 1 Introduction

Oxygen reduction reaction (ORR) is an important process in many electrochemical energy conversion techniques, such as fuel cells and metal–air batteries.^[^ ^1^ ^]^ The ORR reaction occurs at the cathode is a multistep process with slow kinetics, limiting the output power of fuel cells and metal–air batteries.^[^ ^2^ ^]^ Platinum is the highly active commercial ORR electrocatalyst.^[^ ^3^ ^]^ However, platinum‐based catalysts suffer from the poison of methanol and carbon monoxide. The high‐price also impedes its large‐scale utilization in energy conversion technologies.^[^ ^4^ ^]^ Therefore, the exploitation of non‐precious and high‐performance ORR electrocatalysts is vital.^[^ ^5^ ^]^

Over the past years, various non‐precious metal‐based materials including metal‐nitrogen‐carbon, perovskites, transition metal oxides and heteroatom‐doped carbon materials have received wide attention as their low cost and excellent ORR catalytic performance.^[^ ^6^ ^]^ Particularly, transition metal supported on nitrogen doped carbon (M‐N‐C, M = Fe, Co, and Ni, etc.) materials have been widely reported as efficient non‐precious metal‐based ORR electrocatalysts due to their large surface area, flexibilities of the dopant and favorable electrical conductivity.^[^ ^7^ ^]^ Nanoparticles on the nitrogen‐doped carbons are mostly studied, in which the electron transfer can occur between the nanoparticles and the nitrogen‐doped carbon to improve the ORR activity.^[^ ^8^ ^]^ The metal moieties with nanoclusters and even with single‐atom existing form hold great promise in electrocatalysis, which can release active sites to a larger extent.^[^ ^9^ ^]^ Furthermore, introduction of binary active sites is an effective approach to improve the electrocatalytic activity of catalysts due to the synergistic effect between the two species.^[^ ^10^ ^]^ The binary active sites design, such as FeCo and NiFe dual‐doped metal nanoparticles supported on carbon electrocatalysts have been demonstrated to improve the ORR activity.^[^ ^11^ ^]^ However, the structural instability of the carbon materials as a carrier might lead to the agglomeration of metal nanoparticles, and therefore degrades the activity and durability of the catalysts. Accordingly, the development alternative catalyst support with good stability and highly dispersed active sites is urgently acquired.^[^ ^12^ ^]^

Metal oxides^[^ ^13^ ^]^ (WO*_x_*, SnO*_x_*, MoO*_x_*, etc.) are widely used as catalyst support in heterocatalysis. Oxides usually exhibit good stability,^[^ ^14^ ^]^ and more importantly, the formation of oxygen vacancies in metal oxides is not only favorable for the formation of well‐dispersed and highly active metal sites, but also can improve the electrical conductivity of catalysts.^[^ ^15^ ^]^ Meanwhile, oxygen‐deficient sites can also be utilized to tune the surface coordination sates of the supported metal catalysts and enhance the activity of the catalyst. Additionally, the specific area and porosity of the catalyst have great influences on their ORR activity. 3D ordered macropores (3DOM) materials possess various advantages such as large specific surface area, high mass transfer efficiency, good structural stability.^[^ ^16^ ^]^ Therefore, the development of oxygen‐deficient metal oxide supported composite with macroporous architecture can be a promising strategy toward the design of efficient and durable ORR catalyst.

In the present work, an efficient and stable 3DOM structured ORR catalyst is developed, which consists of binary Fe/Co nanoclusters supported on defective tungsten oxide confined with a N‐doped carbon layer (3DOM Fe/Co@NC‐WO_2−_ *_x_*). The 3DOM architecture design not only offers a high surface area to expose active sites, but also accelerates the mass transportation at the solid‐electrolyte‐gas reaction interface. The NC‐WO_2−_ *_x_* support improves the conductivity of the catalyst, and at the same time provides abundant anchored sites for the supported Fe/Co nanoclusters. Raman, electron paramagnetic resonance (EPR) and X‐ray photoelectron spectroscopy (XPS) analysis prove that ammonia treatment can cause more defects compared with the sample without ammonia treatment (referred as to 3DOM Fe/Co@C‐WO_2−_ *_x_*), which promote the ORR activity. Moreover, theoretical calculations show that the synergistic effect of binary Fe and Co on N doped WO_2−_ *_x_* support can promote the energetics of the key ORR elementary reaction compared with the catalysts of monometallic Fe or Co on N‐doped WO_2−_ *_x_* support (named as 3DOM Fe@NC‐WO_2−_ *_x_* and 3DOM Co@NC‐WO_2−_ *_x_*, respectively), and therefore contribute to the high activity of 3DOM Fe/Co@NC‐WO_2−_ *_x_* catalyst. Accordingly, the optimized 3DOM Fe/Co@NC‐WO_2−_ *_x_* delivers a half wave potential (*E*_1/2_) of 0.87 V for ORR, which is equivalent to that of Pt/C, and higher than that of referenced catalysts. Remarkably, the performance of the zinc–air batteries (ZABs) with 3DOM Fe/Co@NC‐WO_2−_ *_x_* cathode is significantly improved, with a specific capacity of 757 mA h g_Zn_^–1^ and a energy density of 968.96 Wh kg^–1^, outperforming the benchmark of Pt/C catalyst.

## 2 Results and Discussion

**Figure 1:**
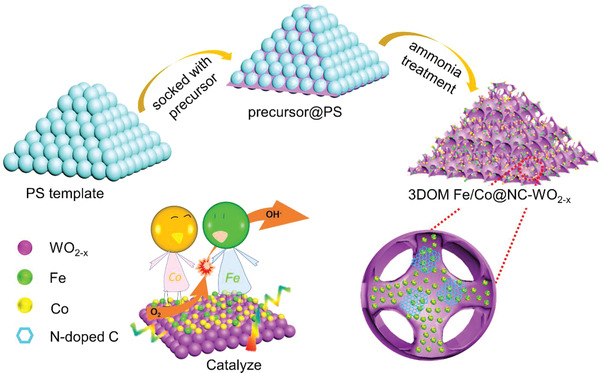
The preparation process of 3DOM Fe/Co@NC‐WO_2−_ *_x_* and the stabilization effect of Fe/Co nanoclusters by NC‐WO_2−_ *_x_*.

**Figure** **1** shows the fabrication process of 3DOM Fe/Co@NC‐WO_2−_ *_x_*, which was prepared by immersing polystyrene (PS) template in a precursor solution followed by calcining process. During the heat and ammonia treatment, the PS template was decomposed and removed to obtain the 3DOM WO_2−_ *_x_* framework and a N‐doped carbonaceous layer (NC), on which Fe/Co nanoclusters was loaded. This NC layer is beneficial to improve the ORR catalytic activity of the catalyst.^[^ ^17^ ^]^ The ammonia treatment also leads to the formation of oxygen vacancies in WO_2−_ *_x_*, which not only enhances the conductivity of this oxide support, but also benefits the dispersion and the stabilization of the supported binary Fe/Co nanoclusters through strong metal‐support interaction. In addition, this ordered macroporous structural design can also accelerates the mass transportation at the solid‐electrolyte‐gas reaction interface, benefiting the ORR electrochemical performance. Accordingly, all of these features would contribute to an enhanced ORR activity of the 3DOM Fe/Co@NC‐WO_2−_ *_x_* catalyst.

**Figure 2:**
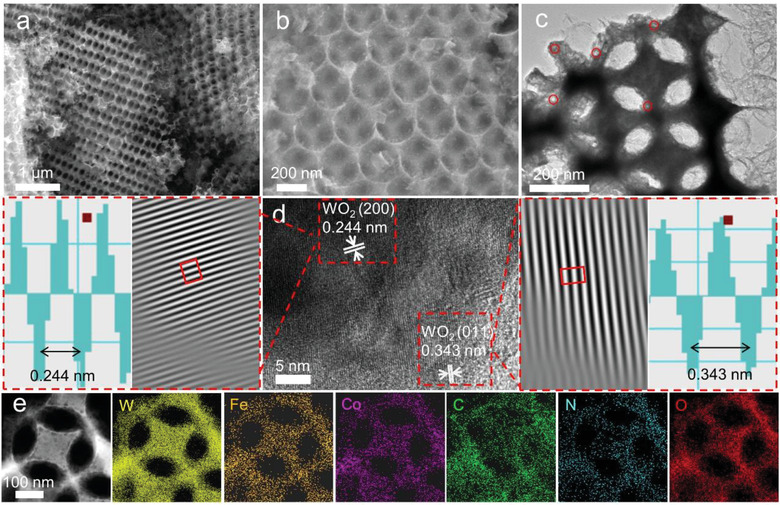
a,b) SEM images, c) TEM image, d) HRTEM image, and e) high‐magnification TEM mapping of the 3DOM Fe/Co@NC‐WO_2−_ *_x_*.

The scanning electron microscope (SEM) image in **Figure** **2**a shows the 3D ordered macroporous morphology of 3DOM Fe/Co@NC‐WO_2−_ *_x_*. In the high‐magnification SEM image (Figure 2b), the highly ordered and interconnected framework between adjacent spherical pores is clearly observed. The high‐magnification transmission electron microscope (TEM) image in Figure 2c shows that the macropores are in the diameter of about 220 nm. In addition, lots of mesopores with size of ≈16 nm in the 3DOM framework also can be clearly observed. The high‐resolution TEM image shows the high crystallinity of WO_2_ with lattice fringes of 0.343 and 0.244 nm, which matches well with the WO_2_ (011) and (200) crystal plane (Figure 2d). The selected area electron diffraction (SAED) pattern of 3DOM Fe/Co@NC‐WO_2−_ *_x_* also proves the crystallized feature of WO_2_ (Figure S1, Supporting Information). Interestingly, Figure S2 in the Supporting Information reveals that the 3DOM Fe/Co@NC‐WO_2−_ *_x_* has an IV type N_2_ adsorption‐desorption isotherm. This shows that, beyond the macropores, mesopores and micropores also exist. The Brunauer–Emmett–Teller (BET) specific surface area was calculated to be 98.40 m^2^ g^–1^. The high surface area together with its hierarchically porous structure endows high accessibility of active sites and mass transportation within the catalysts, which benefit the electrochemical performance of the catalyst. Additionally, the TEM elemental mapping images of 3DOM Fe/Co@NC‐WO_2−_ *_x_* further reveal the uniform distribution of W, Fe, Co, C, N, and O elements in the macroporous framework (Figure 2e).

**Figure 3:**
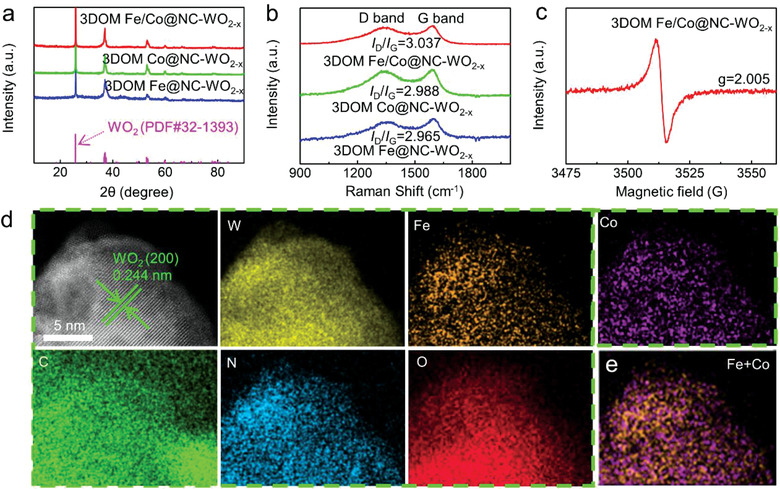
a) XRD patterns, b) Raman spectra, c) EPR spectra, d) The HAADF‐STEM images and corresponding elemental mappings of W, Fe, Co, C, N, and O on WO_2−_ *_x_* crystal, e) HAADF‐STEM elemental mapping overlap of Fe and Co on WO_2−_ *_x_* crystal in 3DOM Fe/Co@NC‐WO_2−_ *_x_*.

The diffraction peaks of catalysts are presented in **Figure** **3**a. The crystal phase of 3DOM Fe/Co@NC‐WO_2−_ *_x_*, 3DOM Co@NC‐WO_2−_ *_x_*, and 3DOM Fe @NC‐WO_2−_ *_x_* are identified as WO_2_. Note that, no other diffraction peaks are observed, suggesting the Fe and Co species are not crystals. 3DOM Fe/Co@C‐WO_2−_ *_x_* (Ar) is composed of WO_2_ and W_18_O_49_ (Figure S3, Supporting Information). The disordered carbon (D‐band) and graphitic carbon (G‐band) intensity ratio (*I*_D_/*I*_G_) in the Raman spectroscopy of 3DOM Fe/Co@NC‐WO_2−_ *_x_* 3DOM Co@NC‐WO_2‐__x_, and 3DOM Fe@NC‐WO_2−_ *_x_* are 3.037, 2.988, and 2.965, respectively (Figure 3b), which are all comparable but higher than that of 3DOM Fe/Co@C‐WO_2−_ *_x_* (Ar) (*I*_D_/*I*_G_ = 2.831) (Figure S4, Supporting Information). This indicates that more carbon defects are formed in 3DOM Fe/Co@NC‐WO_2−_ *_x_*, which is also beneficial for the ORR catalytic performance.^[^ ^15^ ^]^ In addition, the 3DOM Fe/Co@NC‐WO_2−_ *_x_* catalyst shows a symmetrical EPR signal at *g* = 2.005, confirming the existence of oxygen vacancies in this composite (Figure 3c). The intensity of the EPR peaks is a positive correlation with the content of oxygen vacancies. A relatively lower EPR signal is measured for 3DOM Fe/Co@C‐WO_2−_ *_x_* (Ar) (Figure S5, Supporting Information), suggesting more oxygen vacancies was created in 3DOM Fe/Co@NC‐WO_2−_ *_x_* under ammonia treatment. The states of Fe and Co on WO_2−_ *_x_* support and N‐doped carbon layer in 3DOM Fe/Co@NC‐WO_2−_ *_x_* was further determined by aberration‐corrected (AC) high‐angle annular dark‐field scanning transmission electron microscopy (HAADF‐STEM). Figure 3d also shows the clear lattice fringes with spacing of 0.244 nm, which corresponds to the (200) plane of WO_2_. The element mapping image shows that the distribution of N and W elements is basically consistent on WO_2−_ *_x_*, while the distribution of N and C elements is hardly coincident, indicating the successful doping of N in WO_2−_ *_x_*. Importantly, Fe and Co elements are also found uniformly dispersed on WO_2_ lattice. The dense distribution of Fe and Co implies that they are more likely in the form of Fe/Co nanoclusters with diameters of about 0.4 nm rather than single atoms (Figure 3d; Figure S14, Supporting Information). Notably, the overlapped mapping image of Fe and Co elements proves that their distribution positions are similar and mostly overlapped (Figure 3e). This suggests that there may exists charge transfer between them, which is verified in the following DFT calculations. Figure S6 in the Supporting Information presents the AC HAADF‐STEM image of the NC layer in 3DOM Fe/Co@NC‐WO_2−_ *_x_*. The evenly dispersed highly dense bright spots can be also recognized as Fe/Co nanoclusters.

**Figure 4:**
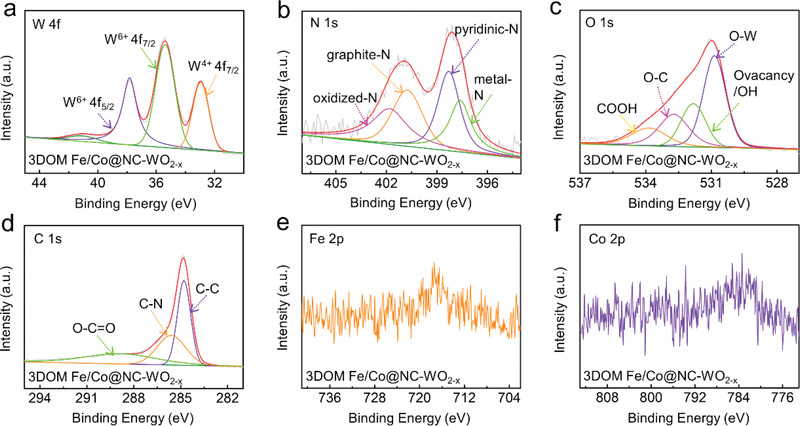
a) W 4f, b) N 1s, c) O 1s, d) C 1s, e) Fe 2p, and f) Co 2p high‐resolution XPS spectra of 3DOM Fe/Co@NC‐WO_2−_ *_x_*.

The surface valence states of the 3DOM Fe/Co@NC‐WO_2−_ *_x_* catalysts were further analyzed by XPS spectrum. For comparison, the XPS spectra of 3DOM Fe/Co@C‐WO_2−_ *_x_* (Ar) is also presented (Figure S7, Supporting Information). **Figure** **4**a is the W 4f XPS spectrum of 3DOM Fe/Co@NC‐WO_2−_ *_x_*. The peaks at 35.8 and 37.8 eV corresponding to W 4f_7/2_ and W 4f_5/2_ of W^6+^, respectively, and the peak at 33.0 eV belongs to W 4f_7/2_ of W^4+^.^[^ ^16^, ^18^ ^]^ The W 4f XPS spectrum exhibits the characteristic peak of W^6+^. This is because the WO_2_ surface can be easily oxidized in air.^[^ ^19^ ^]^ The peak at 34.1 eV of 3DOM Fe/Co@C‐WO_2−_ *_x_* (Ar) is ascribed to W 4f_7/2_ of W^5+^.^[^ ^20^ ^]^ Compared with that of 3DOM Fe/Co@C‐WO_2−_ *_x_* (Ar), the oxidation state of W in 3DOM Fe/Co@NC‐WO_2−_ *_x_* is reduced, suggesting the formation of oxygen vacancy after the ammonia treatment.^[^ ^21^ ^]^ The N 1s spectrum for 3DOM Fe/Co@NC‐WO_2−_ *_x_* was deconvoluted to four peaks located at 397.8, 398.4, 400.6, and 401.8 eV (Figure 4b), which corresponds to metal‐N, pyridinic‐N, graphite‐N, and oxidized‐N, respectively.^[^ ^22^ ^]^ The percentage for oxidized‐N, graphite‐N, pyridinic‐N, and metal‐N are 24.8%, 24.97%, 28.46%, and 21.77%, respectively (Table S2, Supporting Information). Pyridinic‐N is beneficial for the formation of 4‐electron pathway with water as products and can reduces the generation of H_2_O_2_, and graphite‐N facilitates electron transfer from the carbon electronic bands to the antibonding orbitals of oxygen, thus contributing to the catalytic performance of ORR.^[^ ^23^ ^]^ The metal‐N can be index to Fe—N and Co—N bonds, which are recognized to facilitate the catalytic activity of ORR as well.^[^ ^23^ ^]^ In XPS spectra of O 1s, the peaks at 530.8 and 531.8 eV are associated with O—W bond and the non‐lattice (defective) O^2–^ or OH species (Figure 4c). The existence of defective O^2–^ is consistent with the reduced valance state in the W 4f spectrum (Figure 4a), further verifying that 3DOM Fe/Co@NC‐WO_2−_ *_x_* is enriched with oxygen vacancies. The other two peaks of 532.7 and 533.8 eV belong to O—C and COOH, respectively.^[^ ^15^ ^]^ According to Figure 4d, the C 1s peaks at 284.7, 285.7, and 289.1 eV can be assigned to C—C, C—N and O—C═O bonds, respectively.^[^ ^24^ ^]^ As discussed in the previous sections, the Fe and Co are likely in the forms of clusters on WO_2−_ *_x_* support and NC layer. This suggests that the Fe 2p and Co 2p XPS spectrum of 3DOM Fe/Co@NC‐WO_2−_ *_x_* should have a very low signal (Figure 4e,f).

**Figure 5:**
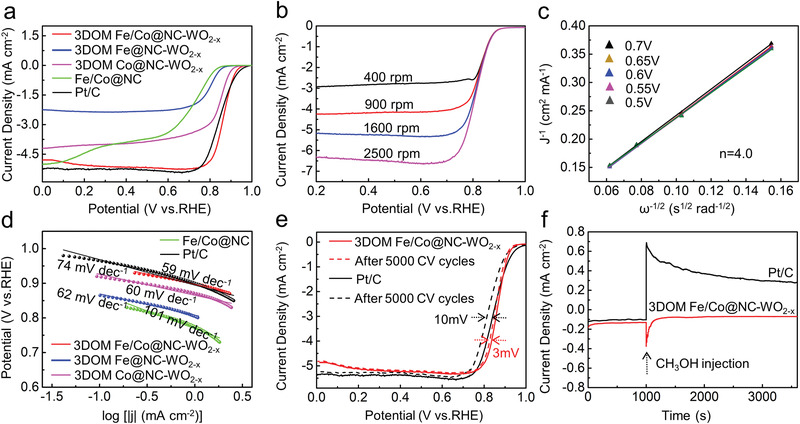
a) LSV curves of the catalysts with a sweep rate of 10 mV s^–1^. b) LSV curves at different rotation speeds. c) K–L plots of the 3DOM Fe/Co@NC‐WO_2−_ *_x_*. d) Tafel plots of 3DOM Fe@NC‐WO_2−_ *_x_*, 3DOM Co@NC‐WO_2−_ *_x_*, Fe/Co@NC, 3DOM Fe/Co@NC‐WO_2−_ *_x_* and Pt/C. e) The LSV curves of 3DOM Fe/Co@NC‐WO_2−_ *_x_* and Pt/C before and after 5000 CV cycles between 0.6 and 1.0 V (vs. RHE) at a speed of 100 mV s^–1^. f) Chronoamperometric response of the 3DOM Fe/Co@NC‐WO_2−_ *_x_* and Pt/C at 0.6 V (vs. RHE) before and after adding 0.5 m CH_3_OH to 0.1 m KOH solution.

The ORR activities of catalysts were studied using linear sweep voltammetry (LSV) in 0.1 m KOH. **Figure** **5**a reveals that, although the onset potential of 3DOM Fe/Co@NC‐WO_2−_ *_x_* (0.93 V) cannot catch up with Pt/C (0.98 V), the half‐wave potential (*E*_1/2_) of 3DOM Fe/Co@NC‐WO_2−_ *_x_* (0.87 V) is 20 mV higher than that of Pt/C (*E*_1/2_ = 0.85 V). Meanwhile, the *E*_1/2_ of 3DOM Fe/Co@NC‐WO_2−_ *_x_* is also higher than those of 3DOM Fe@NC‐WO_2−_ *_x_* (*E*_1/2_ = 0.80 V) and 3DOM Co@NC‐WO_2−_ *_x_* (*E*_1/2_ = 0.83 V). This clearly indicates there is a synthetic effect of binary Fe/Co species promoting the ORR activity. Moreover, the activity of 3DOM Fe/Co@NC‐WO_2−_ *_x_* is also much higher than the Fe/Co@NC (*E*_1/2_ = 0.73 V). This observation implies that the WO_2−_ *_x_* support might plays a more critical role in improving the ORR activity of 3DOM Fe/Co@NC‐WO_2−_ *_x_* than the NC layer in tuning the activity of Fe/Co nanoclusters. The activity of 3DOM Fe/Co@NC‐WO_2−_ *_x_* was also superior than most of the recently reported electrocatalysts (Table S1, Supporting Information). In addition, the higher activity of 3DOM Fe/Co@NC‐WO_2−_ *_x_* compared with 3DOM Fe/Co@C‐WO_2−_ *_x_* (Ar) confirmed the positive effect of N‐doped WO_2−x_ support on ORR performance (Figure S8, Supporting Information). Moreover, the ORR activity of 3DOM Fe/Co@NC‐WO_2−_ *_x_* (10 min NH_3_ and 800 °C treatment) was superior to that of others with different NH_3_ treatment time and annealing temperatures, which provides the most favorable degree of oxygen vacancies and allows for the highest ORR activity. It also demonstrates that the W/Fe/Co molar ratio with 8:1:1 is critical for the optimized activity, which ensures the favorable synthetic effect of binary Fe and Co clusters (Figure S9, Supporting Information). The ORR reaction kinetics of 3DOM Fe/Co@NC‐WO_2−_ *_x_* was further studied using the Koutecky–Levich (K–L) equations based on the LSV curves obtained at various rotating rates (Figure 5b,c). The linear relationship of the K‐L curves shows that ORR is the first order reaction dynamics and the electron transfer number (*n*) is ≈4.0, showing the ORR reaction follows the 4‐electron pathway.^[^ ^7c^ ^]^ Furthermore, the Tafel slope of 3DOM Fe/Co@NC‐WO_2−_ *_x_* is 59 mV dec^–1^, lesser than that of 3DOM Co@NC‐WO_2−_ *_x_* (60 mV dec^–1^), 3DOM Fe@NC‐WO_2−x_ (62 mV dec^–1^), Fe/Co@NC (101 mV dec^–1^), and Pt/C (74 mV dec^–1^), see Figure 5d. The lower Tafel slope of 3DOM Fe/Co@NC‐WO_2−_ *_x_* suggests its faster ORR kinetics than that of monometallic Fe or Co‐based catalysts. This is mainly attributed to that the synthetic effect of the binary metals can lower the energy barriers of the ORR reaction, which is discussed in detail in the following DFT results. These results show that 3DOM Fe/Co@NC‐WO_2−_ *_x_* with hierarchically porous structure and binary Fe/Co active sites has high catalytic activity toward ORR.

In addition to its activity, the durability performance of the catalysts is even more critical for its practical applications. Figure 5e shows that, after 5000 CV cycles of acceleration dynamic test, the negative shift of *E*_1/2_ for 3DOM Fe/Co@NC‐WO_2−_ *_x_* is only 3.0 mV, lower than that of Pt/C (10.0 mV), indicating the excellent stability of 3DOM Fe/Co@NC‐WO_2−_ *_x_* catalyst. In addition, after chronoamperometric stability test for 24 h, the retention rate of current density for 3DOM Fe/Co@NC‐WO_2−_ *_x_* is 98%, higher than that of Pt/C (78%) (Figure S10, Supporting Information), indicating the excellent stability of 3DOM Fe/Co@NC‐WO_2−_ *_x_* catalyst. SEM image and X‐ray diffraction (XRD) pattern of 3DOM Fe/Co@NC‐WO_2−_ *_x_* after long‐time stability test reveal that it still maintains the 3D ordered macroporous structure and the crystalline phase of WO_2_, indicating the stability of the material (Figures S11 and S12, Supporting Information). On the other hand, the methanol tolerance of the catalysts was also examined (Figure 5f). After the CH_3_OH (0.5 m) solution was injected, the current density of the 3DOM Fe/Co@NC‐WO_2−_ *_x_* catalyst has a negligible change compared with Pt/C. These results show the 3DOM Fe/Co@NC‐WO_2−_ *_x_* has outstanding stability and methanol tolerance performance in alkaline solution.

**Figure 6:**
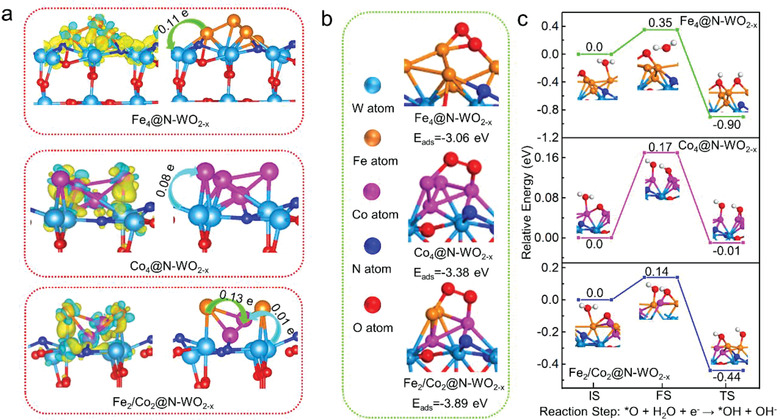
DFT calculation results of a) charge density difference plots and Bader charge transfer analysis in Fe_4_@N‐WO_2−_ *_x_*, Co_4_@N‐WO_2−_ *_x_*_,_ and Fe_2_Co_2_@N‐WO_2−_ *_x_* model; the yellow and cyan color represent the charge accumulation and depletion area; b) O_2_ adsorption configuration and c) optimized structures of the initial state (IS), transition state (TS), and final state (FS) on Fe_4_@N‐WO_2−_ *_x_*, Co_4_@N‐WO_2−_ *_x_* and Fe_2_Co_2_@N‐WO_2−_ *_x_* for the reaction: O* + H_2_O + e^–^ → OH* + OH^–^.

It has been discussed previously that there exists of binary Fe/Co cluster in Fe/Co@NC‐WO_2−_ *_x_*, and the N‐doped WO_2−_ *_x_* support plays a critical role in affecting its ORR activity. Therefore, the synergistic effect of binary Fe/Co cluster on N‐doped WO_2−_ *_x_* (N‐WO_2−_ *_x_*) support was investigated by DFT calculations, in which the Fe_4_@N‐WO_2−_ *_x_*, Co_4_@N‐WO_2−_ *_x_* and Fe_2_Co_2_@N‐WO_2−_ *_x_* models was considered. Bader charge analysis shows that, on Fe_4_@N‐WO_2−_ *_x_*, an average of 0.11 e was transferred from the supported Fe_4_ cluster to the N‐WO_2−_ *_x_* support. On the other hand, on Co_4_@N‐WO_2−_ *_x_*, the Co_4_ cluster gained an average of 0.08 e charge from the N‐WO_2−_ *_x_* support. However, in Fe_2_Co_2_@N‐WO_2−_ *_x_*, the Co atom received 0.01 e charge from N‐WO_2−_ *_x_*, and notably the Co atom gained additional more electrons (0.13 e) from its neighboring Fe atom, which precisely proves that the charge transfer between Fe and Co, consistent with the result in Figure 3e (**Figure** **6**a). That is, in N‐WO_2−_ *_x_* supported binary Fe/Co cluster, the Co acquires additional electrons from Fe, and thus inducing an in‐plane charge redistribution promoting the ORR reaction kinetics. To provide direct support for this argument, some key ORR reaction steps including the O_2_ adsorption and OH* formation steps in ORR reaction pathways were considered.^[^ ^25^ ^]^ Figure 6b shows the O_2_ adsorption configuration and energies on Fe_4_@N‐WO_2−_ *_x_* (−3.06 eV), Co_4_@N‐WO_2−_ *_x_* (−3.38 eV) and Fe_2_Co_2_@N‐WO_2−_ *_x_* (−3.89 eV) model. The highest O_2_ adsorption energy (*E*_ads_) on Fe_2_Co_2_@N‐WO_2−_ *_x_* indicates that the supported binary catalyst is more favorable for O_2_ adsorption compared with that of supported monmetallic Fe or Co catalysts. Moreover, Figure 6c reveals the reaction kinetics for *H_2_O transition into *OH on different catalysts. The activation energies (reaction energies) of this reaction step on Fe_4_@N‐WO_2−_ *_x_*, Co_4_@N‐WO_2−_ *_x_* and Fe_2_Co_2_@N‐WO_2−_ *_x_*, are 0.35 (−0.90), 0.17 (−0.01), and 0.14 (−0.44) eV, respectively. The low energy barrier on Fe_2_Co_2_@N‐WO_2−_ *_x_* suggests that there is a synergistic effect between Fe and Co, promoting the ORR reaction kinetically. This is consistent with the conclusion of Tafel slope discussion.

**Figure 7:**
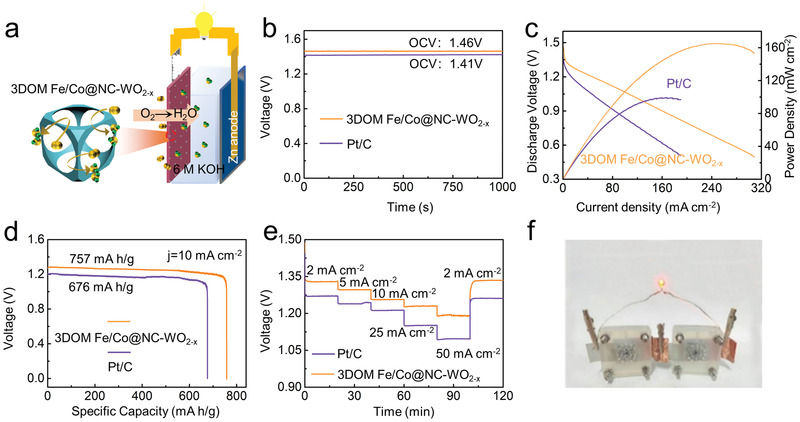
a) Representation of the primary ZAB; b) OCV; c) polarization and power density curves; d) specific capacity results at 10 mA cm^–2^; e) discharging curves at diverse current densities of the ZAB assembled using 3DOM Fe/Co@NC‐WO_2−_ *_x_* and Pt/C catalysts; f) photograph of the LED powered with two 3DOM Fe/Co@NC‐WO_2−x_‐based ZABs connected in series.

To evaluate its practical applied potential, primary ZABs were assembled employing 3DOM Fe/Co@NC‐WO_2−_ *_x_* catalyst (**Figure** **7**a). The benchmark Pt/C is also studied for comparison. The open‐circuit voltage (OCV) of the battery driven by 3DOM Fe/Co@NC‐WO_2−_ *_x_* is 1.46 V, higher than that of Pt/C (1.41 V), demonstrating its excellent electrocatalytic performance (Figure 7b). The peak power density of ZAB with 3DOM Fe/Co@NC‐WO_2−_ *_x_* cathode is 165.1 mW cm^–2^, higher than that of Pt/C (95.5 mW cm^–2^), see Figure 7c. The power density of ZABs with 3DOM Fe/Co@NC‐WO_2−_ *_x_* was compared with the recently reported electrocatalysts (Table S1, Supporting Information). Moreover, the specific capacity of the ZAB at 10 mA cm^–2^ is 757.0 mA h g_Zn_^–1^ based on the standardized weight consumption of zinc, the energy density reaching up to 968.96 Wh kg^–1^, outperforming those of Pt/C (676.0 mA h g_Zn_^–1^ and 811.20 Wh kg^–1^), see Figure 7d. As shown in Figure S13 in the Supporting Information, after long stability test at 10 mA cm^–2^, the zinc–air battery assembled with 3DOM Fe/Co@NC‐WO_2−_ *_x_* has a higher voltage and is more stable than that of Pt/C. The rate performance of the zinc–air battery assembled with 3DOM Fe/Co@NC‐WO_2−_ *_x_* was tested with discharge current density ranging from 2, 5, 10, 25, and 50 mA cm^–2^, finally returning to 2 mA cm^–2^ (Figure 7e). One can see that the voltage retention rate reaches to 99.8% after the discharge current density decreases to the initial value, and even after undergoing high current density of 50 mA cm^–2^. This can be reasonably attributed to that the 3D ordered macroporous structure provides good mass transfer and large specific surface area to expose abundant active sites as well as good structural stability for ORR. Notably, two batteries using 3DOM Fe/Co@NC‐WO_2−_ *_x_* catalyst were connected in series to power the LED (3 V) light, which can be lit up readily, confirming the potential application of the catalyst (Figure 7f).

**Figure 8:**
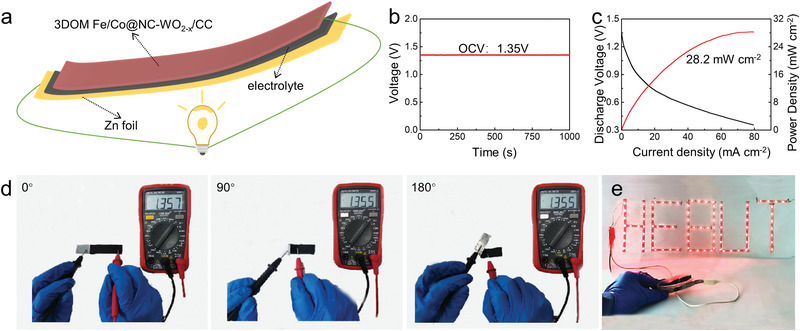
a) A structural representation of the flexible zinc–air battery; b) OCV; c) polarization and power density curves; d) voltage at different bending angles; e) LED lighting demonstration of the flexible zinc–air battery installed using 3DOM Fe/Co@NC‐WO_2−_ *_x_* catalyst.

Given the above excellent performance, flexible zinc–air batteries using the catalyst on the carbon cloth (CC) were prepared (**Figure** **8**a). The battery assembled by 3DOM Fe/Co@NC‐WO_2−_ *_x_* catalyst exhibits a relatively high OCV of 1.35 V, reflecting its excellent ORR activity (Figure 8b). In addition, its peak power density can reach up to 28.2 mW cm^–2^ (Figure 8c), which is comparable with others reported in the literature.^[^ ^26^ ^]^ Different bending conditions of flexible zinc–air battery driven by 3DOM Fe/Co@NC‐WO_2−_ *_x_* were studied to assess the stability of the flexible battery. The high and relatively stable voltage under different bending angle conditions confirms the stable performance of the flexible battery (Figure 8d). Moreover, the flexible zinc air battery in Figure 8e is assembled to power the LED (3 V), which can still work normally under severe bending. All the above studies demonstrate the excellent activity and flexibility of the zinc–air batteries installed by 3DOM Fe/Co@NC‐WO_2−_ *_x_*.

## 3 Conclusion

In the present study, an efficient and durable electrocatalyst of oxygen‐deficient WO_2−_ *_x_* and N‐doped carbon layer supported binary Fe/Co nanocluster catalyst with 3DOM structure design was developed. The 3DOM WO_2−_ *_x_* support obtained under NH_3_ treatment possesses abundant oxygen vacancies and N doping. This not only improves the conductivity and the specific surface area of the catalyst, but also ensures the good dispersion and strong anchor interaction of Fe/Co nanoclusters. Experimental analysis reveals that the synergistic effect of binary Fe and Co on the N‐doped WO_2−_ *_x_* support plays a critical role in governing its ORR activity. This has been further proved by DFT calculations, which shows that there exists electron transfer from both Fe to Co and the support to Fe/Co nanocluster as well in the N‐WO_2−_ *_x_* supported binary Fe/Co cluster, promoting the reaction energetics of critical ORR reaction steps. Consequently, the developed 3DOM Fe/Co@NC‐WO_2−_ *_x_* catalyst delivers an excellent ORR electrocatalytic performance in the alkaline electrolyte. Promisingly, ZAB installed with 3DOM Fe/Co@NC‐WO_2−_ *_x_* cathode exhibits high power density of 165.1 mW cm^–2^ and specific capacity of 757 mA h g_Zn_^–1^. Additionally, the flexible zinc–air battery assembled with 3DOM Fe/Co@NC‐WO_2−_ *_x_* can delivers a peak power density of 28.2 mW cm^–2^, indicating its potential in practical applications. This research opens a novel pathway for the reasonable design of metal oxides supported binary‐metal‐based electrocatalysts with high efficiency and durability for ORR reaction.

## 4 Experimental Section

### Materials and Reagents

Partial ammonium tungstate [(NH_4_)_6_H_2_W_12_O_40_·*x*H_2_O], ferric nitrate [Fe(NO_3_)_3_·9H_2_O, AR, 99%], cobalt nitrate hexahydrate [Co(NO_3_)_2_·6H_2_O, AR, 99%], anhydrous methanol (CH_3_OH), potassium persulfate (K_2_S_2_O_8_), sodium hydroxide (NaOH), polyvinyl pyrrolidone (PVP), styrene (C_8_H_8_), and potassium hydroxide (KOH, AR, 99%) were purchased from Aladdin Industrial Company.

### Material Synthesis

Preparation of polystyrene (PS) template: PS template was synthesized by a simple polymerization reaction.^[^ ^15^ ^]^ First, 45 mL C_8_H_8_ was washed with 10% NaOH solution in a separating funnel, and then washed with distilled water for 4–5 times. Next, 1.2 g PVP was dissolved in 240 mL distilled water and placed in a three‐necked flask protected with Ar gas. After that, 31 mL C_8_H_8_ was added to the three‐necked flask and heated to 75 °C with continuously stirring. Finally, 40 mL K_2_S_2_O_8_ was added to the mixture as the initiator for the polymerization reaction, and stirred for 24 h. After cooling down, the PS emulsion was centrifuged for 16 h at 3500 r min^–1^ and dried at 60 °C.

Preparation of 3DOM Fe/Co@NC‐WO_2−_ *_x_*: 1.49 g (NH_4_)_6_H_2_W_12_O_40_·*x*H_2_O, 0.026 g Fe(NO_3_)_3_·9H_2_O and 0.019 g Co(NO_3_)_2_·6H_2_O with a molar ratio of 8:1:1 were dissolved in a solution containing 2 mL distilled water and 1 mL CH_3_OH. Then, 1 g of PS template was added and soaked in the above solution for 4 h. The resulting suspension was vacuum‐filtered. The filtered sample was dried overnight at room temperature. Then, the solid power was first calcined under Ar atmosphere in a tubular furnace. The temperature was increased to 130 °C kept for 2 h, heated to 500 °C kept for 4 h, and then raised to 800 °C and kept for 30 min. The heating rate of this process is 1 °C min^–1^. After that, the gas was changed to ammonia and kept for 10 min. Then, it was switched back to Ar and cooled down to room temperature. The product was referred as to 3DOM Fe/Co@NC‐WO_2−_ *_x_*. In addition to the 3DOM Fe/Co@NC‐WO_2−_ *_x_* obtained by NH_3_ treatment in 10 min, two other 3DOM samples referred as 3DOM Fe/Co@C‐WO_2−_ *_x_* (Ar) and 3DOM Fe/Co@NC‐WO_2−_ *_x_* (NH_3_ 20 min) were also prepared by changing the duration of NH_3_ treatment. For comparison, samples without the addition of Fe(NO_3_)_3_·9H_2_O, Co(NO_3_)_2_·6H_2_O or (NH_4_)_6_H_2_W_12_O_40_·*x*H_2_O were also synthesized, which were identified as 3DOM Co@NC‐WO_2−_ *_x_*, 3DOM Fe@NC‐WO_2−_ *_x_* and Fe/Co@NC, respectively. Meanwhile, 3DOM composites with different W:Fe:Co molar ratios were also prepared.

### Characterization of Samples

The microstructure of the catalysts was investigated by SEM (Quanta 450 FEG) and TEM (JEOL 2010F). HAADF‐STEM measurements were obtained in an FEI Titan Cubed Themis G2 300 transmission electron microscopy operated at an acceleration voltage of 300 kV and equipped with double spherical aberration (C_s_) correctors. The crystal structure was studied by XRD (D8 Discover). The Bruker A300 EPR was used to obtain the EPR spectrum in the X‐band (9 GHz) at room temperature. The superficial chemical component and the elemental valence state of the catalysts were characterized by XPS (ESCALAB 250 Xi). Raman spectroscopy was recorded on Via Reflex instrument. BET (Autosorb iQ‐MP‐MP) adsorption isotherm was used to calculate the specific surface area.

### Electrochemical Measurements

The electrochemical performance of the catalysts was tested by the rotary disk electrode (RDE, Physicochemical Company, Hong Kong). Volt‐ampere test was conducted on a CHI 760E workstation. In a traditional three‐electrode system, glass carbon rotating disc electrode (5 mm in diameter) coated with 3DOM catalysts was used as the working electrode. Graphite rod was used for counter electrode and Ag/AgCl as reference electrode. The ORR performance of 3DOM catalysts was tested in 0.1 m KOH solution. Details on the preparation method of work electrode are presented in the Supporting Information.

### Density Functional Theory (DFT) Calculation

All the DFT calculations were performed within the generalized gradient (GGA) and Perdew–Burke–Ernzerhof (PBE) functional implemented in VASP code.^[^ ^27^ ^]^ The electron‐ion interaction is described using the projector‐augmented plan wave (PAW) scheme. A cutoff energy of 400 eV for kinetic plane‐wave was applied and a Monkhorst‐Pack 2 × 2 × 1 k‐point mesh was used for integration of the Brillouin zone. The WO_2−_ *_x_* support was constructed on the basis of the WO_2_ (2 × 2) model by removing one surface O atom. The N doped WO_2−_ *_x_* model was built by replacing some of the surface O atom with N on WO_2−_ *_x_*. The supported Co/Fe nanocluster was molded by a cluster with four metal atoms.^[^ ^28^ ^]^ During the optimization, the bottom atomic layer of the models was fixed, whereas the reminding atoms were allowed to relax. The energy convergence is set to 1.0 × 10^–5^ eV per atom and the force convergence is −0.03 eV Å^−1^. The transition state (TS) of the water dissociation reaction was identified using the climbing‐image nudged‐elastic band (CI‐NEB) method.^[^ ^29^ ^]^

### Zinc–Air Battery (ZAB) Tests

The catalyst ink was prepared by dispersing 13.5 mg catalyst into 20 mL isopropanol solution containing 5 wt% nafion and ultrasonicated for 30 min. Then, the catalysts ink was sprayed onto carbon fiber paper and used as cathode. The catalyst loading was 1.0 mg cm^–2^. The Pt/C cathode was prepared in the same method. The loading was 1.0 mg cm^–2^. Polished zinc foil and 6.0 m KOH solution were used as anode and electrolyte, respectively. Polarization curves were tested on a CS2350 station. The discharge curves and the rate performance were tested on the LANHE CT2001A system.

### Flexible Batteries Tests

The method of preparing catalyst ink is the same as that of zinc–air battery. The carbon cloth (CC) was soaked overnight in catalyst ink and then removed to dry. The flexible battery was assembled by CC loaded with catalyst used as the cathode, zinc foil as the anode and a filter paper impregnated with 6.0 m KOH solution as the separator. The open circuit voltage (OCV) and polarization curves of the battery were tested on a CS2350 station.

## Conflict of Interest

The authors declare no conflict of interest.

## Supporting information

Supporting Information

## Data Availability

The data that support the findings of this study are available from the corresponding author upon reasonable request.
