# Supplementary material for: Synergistic Binary Fe–Co Nanocluster Supported on Defective Tungsten Oxide as Efficient Oxygen Reduction Electrocatalyst in Zinc‐Air Battery
Source: Adv Sci (Weinh). 2021 Dec 1;9(4):2104237. doi: 10.1002/advs.202104237 (PMC8811830; doi:10.1002/advs.202104237)
Supplement: Supplementary file 1 — Supporting Information [file ADVS-9-2104237-s001.pdf]

ADVANCED SCIENCE
Open Access

Supporting Information

for *Adv. Sci.*, DOI: 10.1002/advs.202104237

Synergistic Binary Fe-Co Nanocluster Supported on Defective Tungsten Oxide as Efficient Oxygen Reduction Electrocatalyst in Zinc-air Battery

Qinglin Han, Ximeng Zhao, Yuhong Luo, Lanlan Wu, Shujuan Sun, Jingde Li, Guihua Liu*, Zhongwei Chen**

Supporting Information

Synergistic Binary Fe-Co Nanocluster Supported on Defective Tungsten Oxide as Efficient Oxygen Reduction Electrocatalyst in Zinc-air Battery

*Qinglin Han [1], Ximeng Zhao [1], Yuhong Luo [1], Lanlan Wu [1, *], Shujuan Sun [1], Jingde Li [1], Guihua Liu [1, *], Zhongwei Chen [2, *]*

Q. Han, X. Zhao, Y. Luo, Prof. L. Wu, Prof. S. Sun, Prof. J. Li, Prof. Y. Wang, Prof. G. Liu
Hebei Provincial Key Laboratory of Green Chemical Technology and Highly Efficient Energy Saving, Tianjin Key Laboratory of Chemical Process Safety, National-Local Joint Engineering Laboratory for Energy Conservation of Chemical Process Integration and Resources Utilization, School of Chemical Engineering and Technology,
Hebei University of Technology, Tianjin, 300130, P. R China
Prof. Z. Chen
Department of Chemical Engineering, University of Waterloo, Waterloo, ON, Canada N2L 3G1
E-mail: wulanlan@hebut.edu.cn; guihualiu@hebut.edu.cn; zhwchen@uwaterloo.ca

Preparation of catalyst.

The preparation method of the working electrode was as follows: the 3DOM Fe/Co@NC-WO_{2-x} catalyst prepared over 2 mg and 1 mg Vulcan XC-72 ultrasonic were mixed in 1000 μL isopropanol to obtain the catalyst ink. Then 30 μL of ink was dropped onto a newly polished glass carbon electrode and dried at room temperature. The loading for the catalysts at the working electrode is 0.306 mg cm^{-2}. The preparation and loading of Pt/C working electrode are same as that the prepared catalysts. Before data collection, the electrolyte was purified with ultra-pure nitrogen for 30 min. When O_2 and N_2 saturation was 0.1 M KOH, the scanning rate was 50 mV s^{-1}, and the cyclic voltammogram (CV) was obtained. Linear scanning voltammetry (LSV) has a scanning rate of 10 mV s^{-1} and a scanning rate of 1600 rpm.

Electrochemical experiments were carried out at room temperature (25 ± 10 °C). The ORR activity of commercial noble metal catalysts Pt/C was determined by the same method. The load of the commercial Pt/C catalysts is a common reference load in the literature, and the catalytic performance measured in this work is consistent with the results reported in the literature. The current density was normalized to the geometric surface area and all potentials were transformed to reversible hydrogen electrodes according to the equation:

$$E_{RHE} = E_{Ag/AgCl} + 0.205 + 0.059pH \quad (1)$$

The kinetics of the 3DOM Fe/Co@NC-WO_{2-x} was studied by a series of LSV tests at a rate of 10 mV s^{-1}, with the speed varying from 400 rpm to 2500 rpm. In other words, according to the given Koutechy-Levich (K-L) equation, the number of ORR transferred electrons on the 3DOM Fe/Co@NC-WO_{2-x} electrode was determined:

$$\frac{1}{J} = \frac{1}{J_L} + \frac{1}{J_K} = \frac{1}{B\omega^{\frac{1}{2}}} + \frac{1}{J_K} \quad (2)$$

$$B = 0.62nFC_0(D_0)^{\frac{2}{3}}v^{-\frac{1}{6}} \quad (3)$$

Where J is the measured current density, J_L is the diffusion-limiting current density, J_K is the kinetic current density, ω is the electrode rotation rate (rad s^{-1}), n is transferred electron number, F is Faraday constant (96485 C mol^{-1}), C_0 is the bulk concentration of O_2, D_0 is the diffusion coefficient of O_2 and υ is the kinetic viscosity of the electrolyte. For the case of 0.1 M KOH at room temperature, the constants adopted are as follows: $C_0 = 1.2\times10^{-3}$ mol L^{-1}, $D_0 = 1.9\times10^{-5}$ cm^2 s^{-1}, $\upsilon = 0.01$ cm^2 s^{-1}.

Tafel slope is an important parameter for studying reaction mechanism and evaluating catalytic activity. With the increase of overpotential, the lower the slope,

the faster the oxygen production rate. It is calculated by the Tafel equation:[1]

$$\eta = b\log j + a \tag{4}$$

Where j is the current density and b is the slope.

The power density P of zinc air battery is calculated as follows:[2]

$$P = I * V \tag{5}$$

where I is the discharge current density and V is the corresponding voltage.

The energy density is calculated based on applied current I, average discharge voltage V, service time Δt, and weight of zinc consumed W_{Zn}:[3]

$$\text{Energy density (W h kg}^{-1}\text{)} = \frac{I*V*\Delta t}{W_{Zn}} \tag{6}$$

The specific capacity calculation formula is as follows:[4]

$$\text{Specific capacity (mA h g}_{\text{Zn}}^{-1}\text{)} = \frac{I*\Delta t}{W_{Zn}} \tag{7}$$

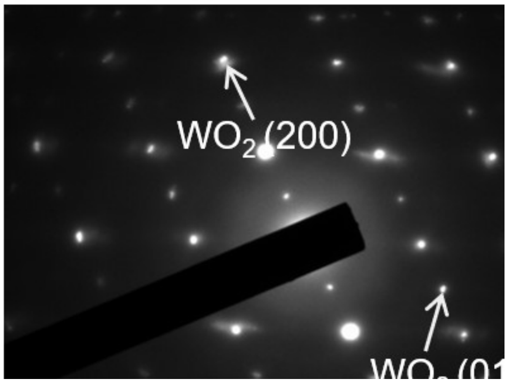

Figure S1. SAED of 3DOM Fe/Co@NC-WO_{2-x}.

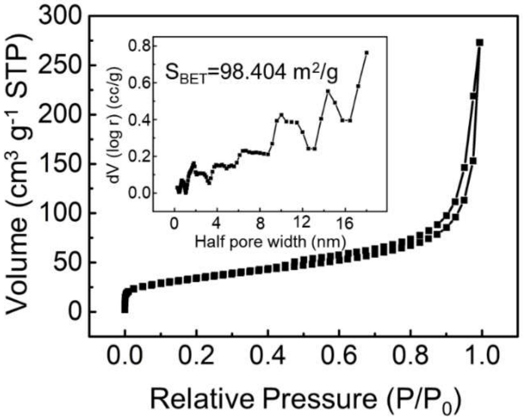

Figure S2. N_2 adsorption-desorption isotherms and pore size distribution of 3DOM Fe/Co@NC-WO_{2-x}.

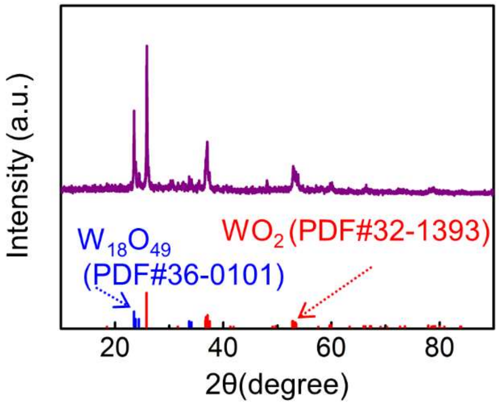

Figure S3. XRD pattern of 3DOM Fe/Co@C-WO_{2-x}.

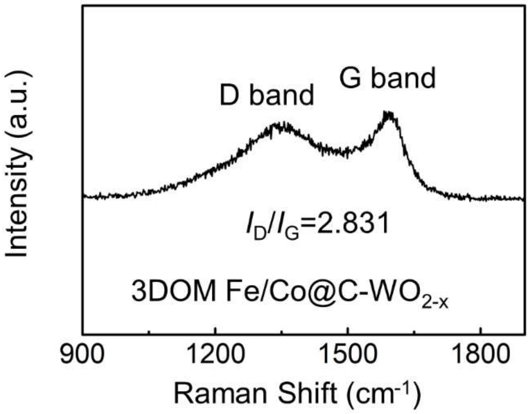

Figure S4. Raman spectra of 3DOM Fe/Co@C-WO_{2-x}.

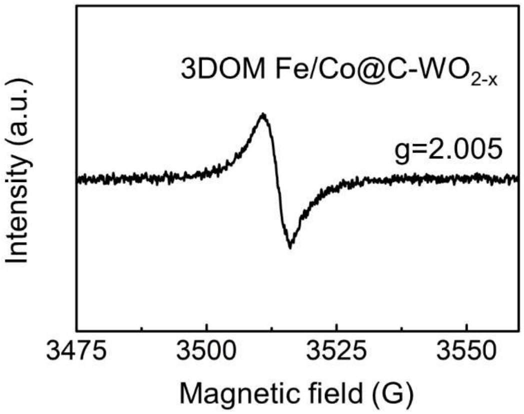

Figure S5. EPR spectra of 3DOM Fe/Co@C-WO_{2-x}.

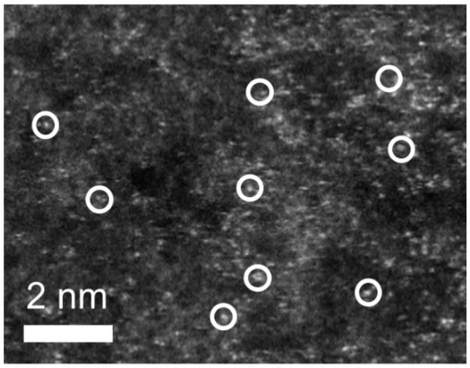

Figure S6. The NC layer in 3DOM Fe/Co@NC-WO_{2-x}.

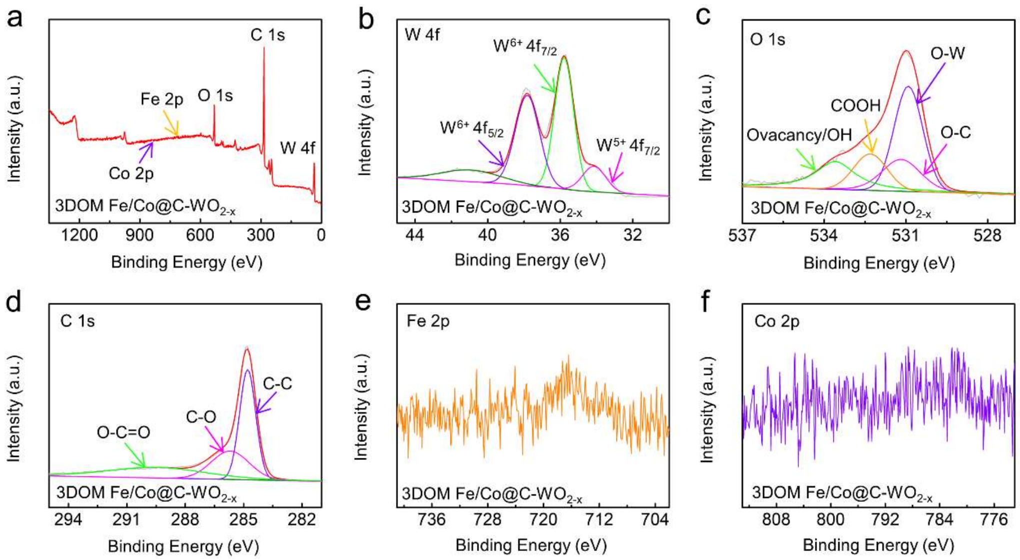

Figure S7. (a) XPS survey spectra, (b) W 4f, (c) O 1s, (d) C 1s, (e) Fe 2p and (f) Co 2p high-resolution XPS spectra of 3DOM Fe/Co@C-WO_{2-x}.

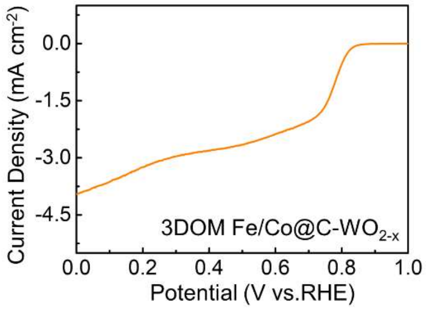

Figure S8. LSV curve of the 3DOM Fe/Co@C-WO_{2-x}.

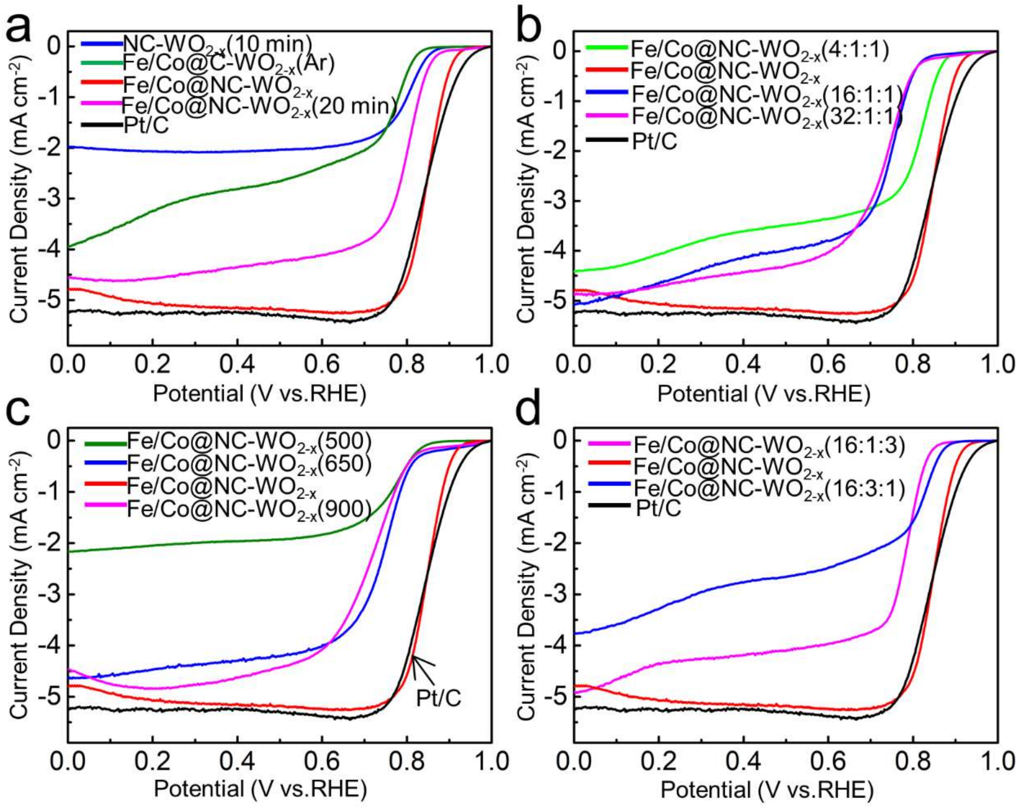

Figure S9. LSV curves of Pt/C and (a) 3DOM composites with different NH_3 treatment time and without Fe and Co addition, (b) 3DOM composites with different W/(Fe+Co) molar ratios, (c) 3DOM composites with different annealing temperatures and (d) 3DOM composites with different Fe/Co molar ratios.

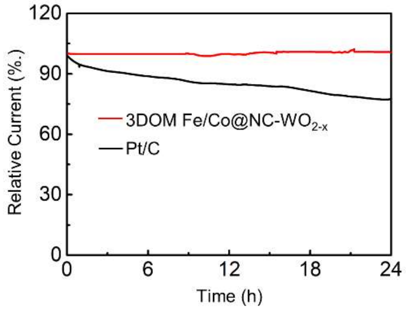

Figure S10. Time dependent chronoamperometric test at 0.6 V (vs. RHE) of 3DOM Fe/Co@NC-WO_{2-x} and Pt/C.

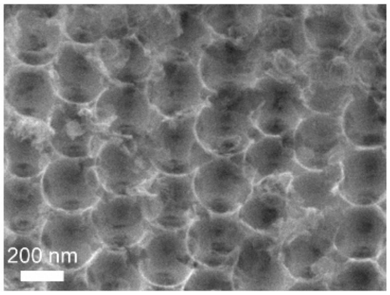

Figure S11. SEM image of 3DOM Fe/Co@NC-WO_{2-x} after chronoamperometric

stability test.

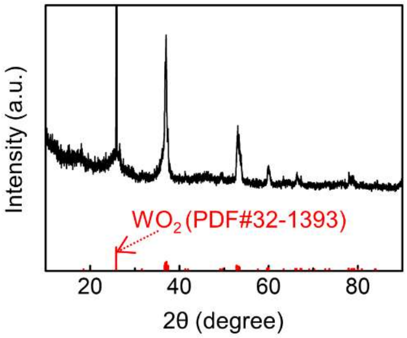

Figure S12. XRD pattern of 3DOM Fe/Co@NC-WO_{2-x} after chronoamperometric stability test.

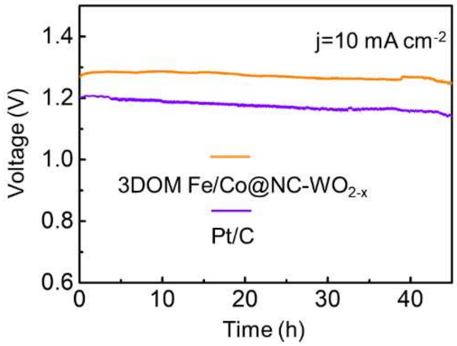

Figure S13. Discharge curves at 10 mA·cm^{-2} for ZABs assembled with 3DOM Fe/Co@NC-WO_{2-x} and Pt/C, respectively.

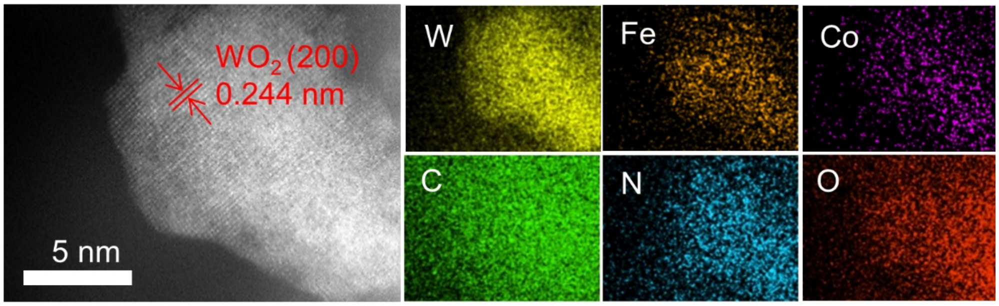

Figure S14. The HAADF-STEM images and corresponding elemental mappings of W, Fe, Co, C, N and O on WO_{2-x} crystal.

Table S1. Comparison of ORR electrocatalytic activity and zinc-air battery performance of 3DOM Fe/Co@NC-WO_{2-x} with reported electrocatalysts.

Electrocatalysts	RDE Loading (mg/cm^2)	$E_{1/2}$ (V vs RHE)	Power density (mW cm^{-2})	specific capacity (mA h g_{Zn}^{-1})	References
3DOM Fe/Co @NC-WO_{2-x}	0.306	0.870	165.1	757.0	This work
$CoMn_2O_4$-S_2	0.320	0.760	108.3	808.2	5
ZFN-900	0.765	0.850	115.8	/	6
Co_3HITP_2	/	0.800	164	784.0	7
NDGs-800	0.204	0.850	115.2	/	8
Pd-Au/Go-4	0.330	0.900	187	/	9
Cu@Fe-N-C	0.500	0.892	92	/	10
Cu/G	0.400	0.850	95.3	/	11
Co-NCF	0.420	0.830	134	766.6	12
GSC-900	0.204	0.827	95	685.0	13
Co@Co_3O_4-1000	0.360	0.800	64	685.0	14
Co/S/N-800	/	0.830	76	805.0	15
Co@NGC-NSs	0.333	0.820	52	743.0	16
Ni-Co-S/NSC	0.350	0.810	137	/	17
(3D) Co-N-C	0.153	0.830	138	/	18
N-$CoSe_2$/3D-MXenx	/	0.790	130	751.0	19
Ni_3Fe/Co-N-C	0.500	0.830	72	/	20

Table S2. The percentage of different N species in the XPS spectrum of N 1s.

N 1s	oxidized-N	graphite-N	pyridinic-N	metal-N
Relative percentage (%)	24.8	24.97	28.46	21.77

Reference

[1] H. Wu, J. Wang, J. Yan, Z. Wu, W. Jin, *Nanoscale* **2019**, *11*, 20144.

[2] L. Ma, S. Chen, Z. Pei, Y. Huang, G. Liang, F. Mo, Q. Yang, J. Su, Y. Gao, J. A. Zapien, C. Zhi, *ACS Nano* **2018**, *12*, 1949.

[3] Y. Guo, P. Yuan, J. Zhang, H. Xia, F. Cheng, M. Zhou, J. Li, Y. Qiao, S. Mu, Q. Xu, *Adv. Funct. Mater.* **2018**, *28*, 1805641.

[4] a) Q. Wang, Y. Ji, Y. Lei, Y. Wang, Y. Wang, Y. Li, S. Wang, *ACS Energy Lett.* **2018**, *3*, 1183; b) S. Ramakrishnan, J. Balamurugan, M. Vinothkannan, A. R. Kim, S. Sengodan, D. J. Yoo, *Appl. Catal. B.* **2020**, *279*, 119381.

[5] Z. Zhang, H. Sun, J. Li, Z. Shi, M. Fan, H. Bian, T. Wang, D. Gao, *J. Power Sources* **2021**, *491*, 229584.

[6] A. Radwan, H. Jin, B. Liu, Z. Chen, Q. Wu, X. Zhao, D. He, S. Mu, *Carbon* **2021**, *171*, 368.

[7] Y. Lian, W. Yang, C. Zhang, H. Sun, Z. Deng, W. Xu, L. Song, Z. Ouyang, Z. Wang, J. Guo, *Angew. Chem. Int. Ed.* **2020**, *59*, 286.

[8] H. Wu, X. Jiang, Y. Ye, C. Yan, S. Xie, S. Miao, G. Wang, X. Bao, *J. Energy Chem.* **2017**, *26*, 1181.

[9] W. Zhu, H. Yuan, F. Liao, Y. Shen, H. Shi, Y. Shi, L. Xu, M. Ma, M. Shao, *Chem. Eng. J.* **2020**, *389*, 124240.

[10] Z. Wang, H. Jin, T. Meng, K. Liao, W. Meng, J. Yang, D. He, Y. Xiong, S. Mu, *Adv. Func. Mater.* **2018**, *28*, 1802596.

[11] G. Han, Y. Zheng, X. Zhang, Z. Wang, Y. Gong, C. Du, M. N. Banis, Y.-M. Yiu,

T.-K. Sham, L. Gu, Y. Sun, Y. Wang, J. Wang, Y. Gao, G. Yin, X. Sun, *Nano Energy* **2019**, *66*, *10*4088.

[12] L. Gao, M. Zhang, H. Zhang, Z. Zhang, *J. Power Sources* **2020**, *450*, 227577.

[13] Z. Ma, K. Wang, Y. Qiu, X. Liu, C. Cao, Y. Feng, P. Hu, *Energy* **2018**, *143*, 43.

[14] Z. Guo, F. Wang, Y. Xia, J. Li, A. G. Tamirat, Y. Liu, L. Wang, Y. Wang, Y. Xia, *J. Mater. Chem. A* **2018**, *6*, 1443.

[15] N. Jia, J. Liu, Y. Gao, P. Chen, X. Chen, Z. An, X. Li, Y. Chen, *ChemSusChem* **2019**, *12*, 3390.

[16] P. Thakur, M. Yeddala, K. Alam, S. Pal, P. Sen, T. N. Narayanan, *ACS Appl. Energy Mater.* **2020**, *3*, 7813.

[17] Z. Wu, H. Wu, T. Niu, S. Wang, G. Fu, W. Jin, T. Ma, *ACS Sustain. Chem. Eng.* **2020**, *8*, 9226.

[18] R. Wang, J. Cao, S. Cai, X. Yan, J. Li, W. M. Yourey, W. Tong, H. Tang, *ACS Appl. Energy Mater.* **2018**, *1*, 1060.

[19] Z. Zeng, G. Fu, H. B. Yang, Y. Yan, J. Chen, Z. Yu, J. Gao, L. Y. Gan, B. Liu, P. Chen, *ACS Mater. Lett.* **2019**, *1*, 432.

[20] J. Tan, T. Thomas, J. Liu, L. Yang, L. Pan, R. Cao, H. Shen, J. Wang, J. Liu, M. Yang, *Chem. Eng. J.* **2020**, *395*, 125151.
